# Multidisciplinary Checklist for Safe Transitions to Home Total Parenteral Nutrition From Hospital-Based Palliative Care: A Case Study From Al Ain, Abu Dhabi

**DOI:** 10.7759/cureus.43350

**Published:** 2023-08-11

**Authors:** Nandan M Shanbhag, Dina F Aljawamis, Mehad Araki, Noor A Abdelbari

**Affiliations:** 1 Oncology/Palliative Care, Tawam Hospital, Al Ain, ARE; 2 Oncology/Radiation Oncology, Tawam Hospital, Al Ain, ARE; 3 Internal Medicine, United Arab Emirates University, Al Ain, ARE; 4 Therapeutics, Tawam Hospital, Al Ain, ARE; 5 Nursing, Tawam Hospital, Al Ain, ARE

**Keywords:** comprehensive care, quality of life, patient outcomes, multidisciplinary team, elderly patients, home total parenteral nutrition (htpn), palliative care

## Abstract

We present a complex case of a multimorbid elderly patient admitted with septic shock, suspected to be secondary to aspiration pneumonia, who subsequently developed an intestinal obstruction due to an ileocecal junction mass. Despite conservative management, the patient's clinical status deteriorated and required comprehensive palliative care. This case highlights the challenges in managing patients with multimorbidities, the importance of a multidisciplinary approach, and the central role of palliative care in the setting of advanced disease. We demonstrate the effectiveness of the above method to safely transit an elderly male with a recent diagnosis of colon cancer with malignant intestinal obstruction, initiated on total parenteral nutrition (TPN).

This study emphasizes the successful implementation of an innovative, multidisciplinary checklist for managing elderly palliative care patients on home total parenteral nutrition (HTPN) in Al Ain, Abu Dhabi. The collaborative approach adopted by the multidisciplinary team (MDT), coupled with comprehensive staff training, patient and caregiver education, and ongoing monitoring and support, facilitated the seamless integration of HTPN into the patient's care plan. The positive outcomes observed in this case underscore the potential of such tailored interventions to bridge the existing gap in HTPN implementation within the region, thus improving the quality of life and overall well-being of elderly patients requiring specialized nutrition support.

## Introduction

The global population is ageing rapidly, with the number of individuals aged 60 and above projected to reach 2.1 billion by 2050, a significant increase from 962 million in 2017 [1]. As the proportion of older adults grows, so does the need for comprehensive and appropriate palliative care to address their complex medical, psychosocial, and emotional needs. Palliative care is particularly important for the elderly, as they often have multiple chronic conditions, decreased functional capacity, and an increased risk of hospitalization [2]. By focusing on symptom management, quality of life, and patient and family support, palliative care can help to alleviate suffering and improve the overall well-being of elderly patients and their caregivers [3].

Home total parenteral nutrition (HTPN) is a specialized form of nutrition support that delivers essential nutrients directly into the bloodstream, bypassing the gastrointestinal tract, for patients with compromised digestive systems or those unable to meet their nutritional needs through oral or enteral routes [4]. By allowing patients to receive nutrition support in the comfort of their homes, HTPN has improved patient outcomes and enhanced the quality of life through increased independence, reduced hospitalization, and greater satisfaction with care [5].

In Al Ain, Abu Dhabi, the implementation of HTPN has been limited, creating a gap in comprehensive care for patients who could benefit from this specialized nutrition support. There is a pressing need to develop tailored approaches and resources to bridge this gap and enhance the quality of life for patients in the region requiring HTPN.

The aim of this study is to present the development and implementation of an innovative, multidisciplinary checklist for managing an elderly palliative care patient on HTPN in Al Ain, Abu Dhabi.

## Case presentation

An 82-year-old patient, with a known case of coronary artery disease (CAD) with a previous history of non-ST segment elevation myocardial infarction (NSTEMI) status post percutaneous coronary intervention (PCI), Alzheimer's dementia, depression, hypertension, chronic kidney disease (CKD) stage 3, dyslipidemia, benign prostate hypertrophy, anemia, vitamin D deficiency, and anal fissure, presented to the emergency department with fever, chills, and productive cough. He also had a newly diagnosed colonic mass, colonic cancer characterized as a signet ring cell carcinoma.

Clinical findings

On examination, the patient was semiconscious and not oriented to time or place and exhibited an Eastern Cooperative Oncology Group performance status of 4 [6]. His physical examination revealed diminished breath sounds in the lower lung fields bilaterally, a soft abdomen with absent bowel sounds, and a percutaneous endoscopic gastrostomy (PEG) tube in situ. No peripheral edema was noted.

Hospital course

**Figure 1:**
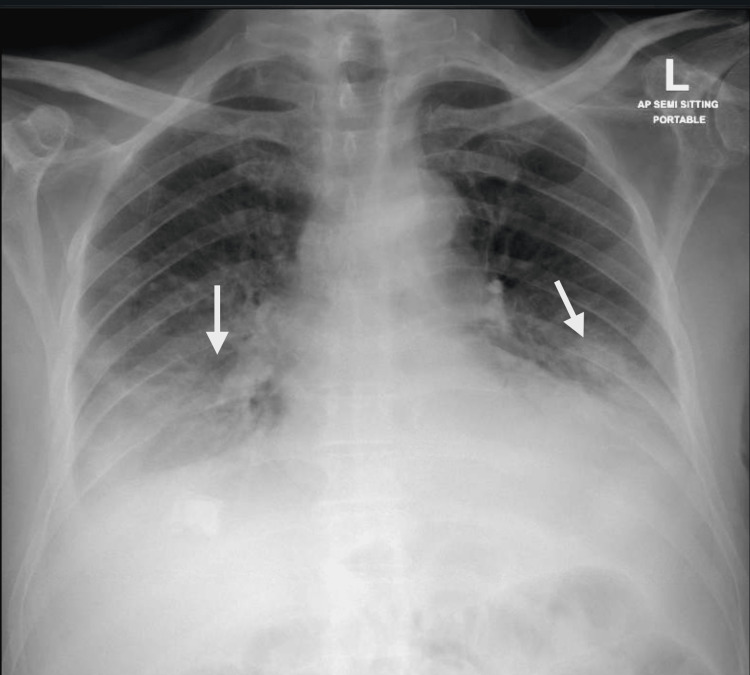
Chest X-ray showing the septal haziness (white arrows)

Upon admission, the patient presented with septic shock. A chest radiograph revealed septal haziness, consistent with aspiration pneumonia (Figure 1).

Management included antibiotic therapy, intravenous (IV) fluids, and hydrocortisone. Due to suspected aspiration pneumonia, the patient's feedings were initially withheld and later resumed with careful transition to continuous feeding.

The patient's condition initially improved; however, he experienced desaturation and cough exacerbation, prompting a switch back to initial antibiotic therapy due to suspected nosocomial pneumonia and aspiration risk. The patient's PEG tube feeding was again withheld due to episodes of vomiting and aspiration.

**Figure 2:**
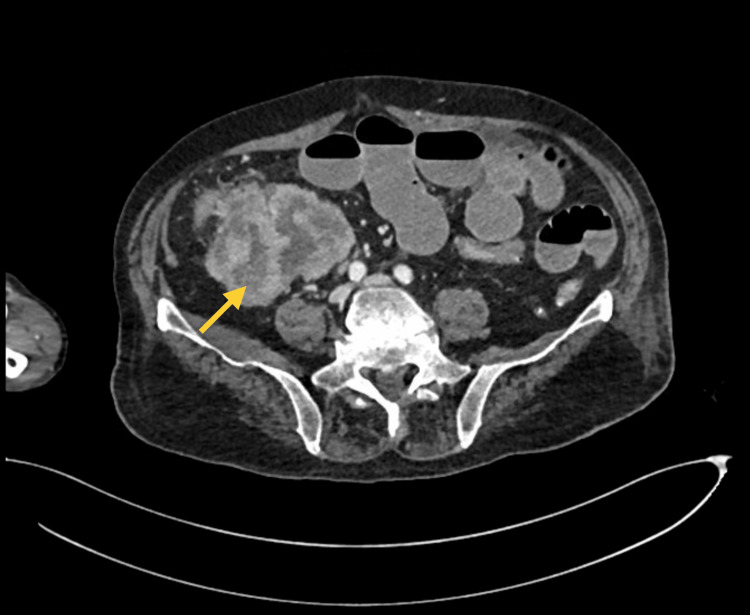
CT scan with contrast showing the mass in the ileocecal junction (yellow arrow) CT: computed tomography

An abdominal radiograph revealed multiple air-fluid levels and small bowel dilatation. A contrast-enhanced computed tomography (CT) scan of the abdomen showed an obstructing mass at the ileocecal junction, leading to the consultation of the general surgery team (Figure 2).

Despite their advice for surgical intervention, the patient's family declined surgery, leading to the initiation of total parenteral nutrition (TPN) and conservative management with PEG tube suction.

The patient later developed recurrent sepsis, likely due to bowel perforation or related complications. His clinical condition continued to deteriorate despite management, leading to consultation with the palliative care team.

Diagnostic assessment

**Figure 3:**
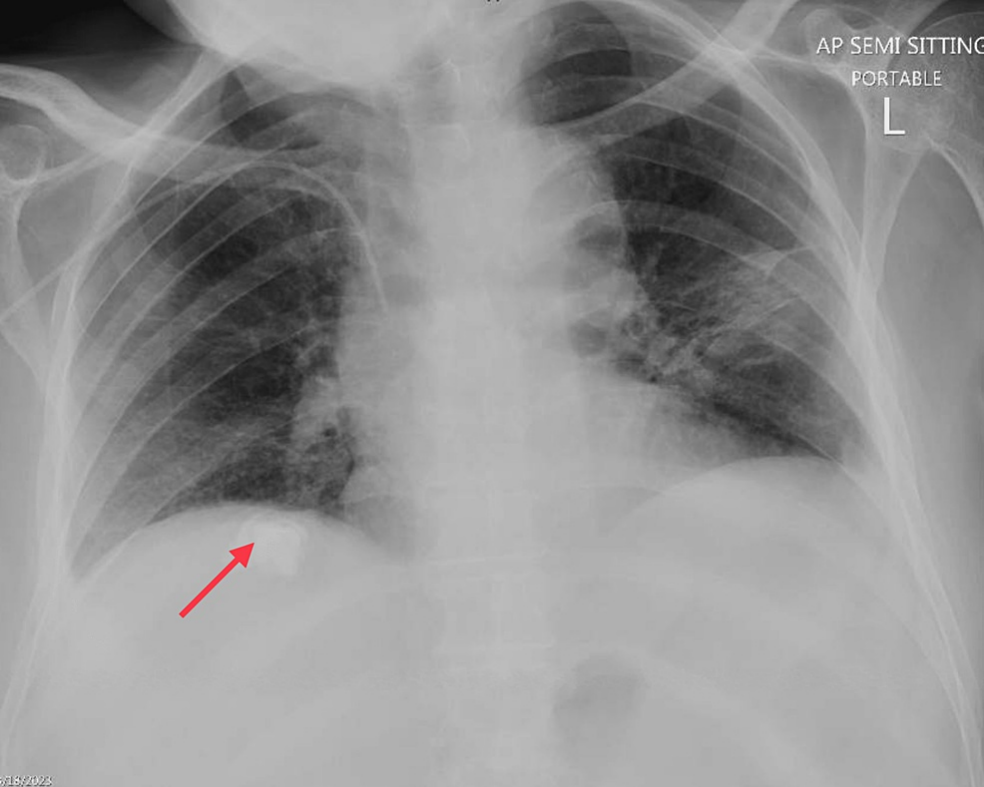
Chest X-ray showing the calcified lesion in the right upper quadrant (red arrow)

**Figure 4:**
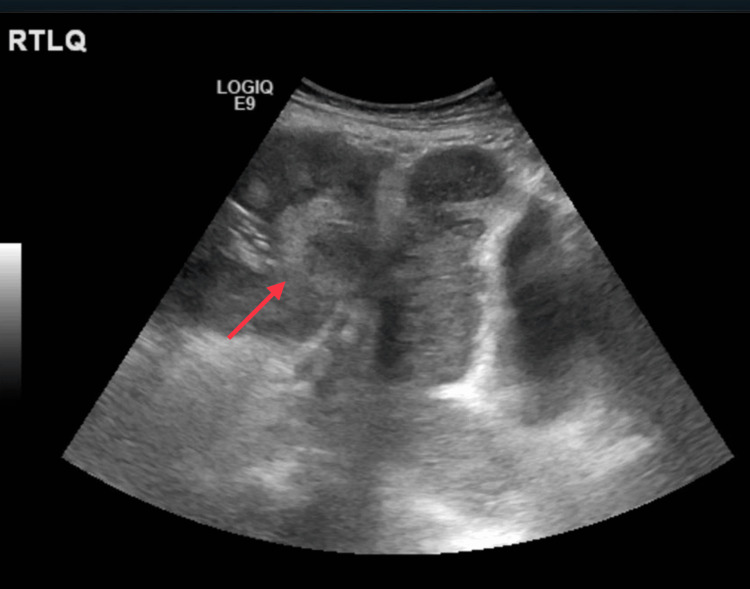
Abdominal ultrasound showing the mass in the right upper quadrant (red arrow)

Blood investigations showed chronic anemia due to malignancy, with normal white blood cells and platelets. Blood glucose levels were around 10 mmol/L. Chest X-ray revealed a calcified hepatic lesion, and abdominal ultrasound showed a mass in the right lower quadrant (Figures 3, 4).

A CT scan of the chest, abdomen, and pelvis with contrast demonstrated a lobulated heterogenous mass at the ileocecal junction and small bowel dilatation, indicative of intestinal obstruction (Figure 2). The patient also had an atrophied left kidney with a prominent collecting system and multiple cortical cysts in the right kidney.

Therapeutic intervention

The patient's treatment plan included comprehensive palliative care, regular chest physiotherapy, pain management, regular medication review for comorbidities, and close monitoring of electrolyte levels, renal function, and nutritional requirements. PEG tube feeding was attempted twice but was unsuccessful due to recurrent vomiting. The patient's anemia was managed with blood transfusions as indicated by hemoglobin levels and clinical condition.

Home TPN

The patient's clinical condition deteriorated despite comprehensive palliative and supportive care. Ongoing discussions were held with the patient's family to address their concerns, answer questions, and provide emotional support. A decision was made to continue the patient on total parenteral nutrition. As the family desired that the patient spend some time at home with family and friends, a multidisciplinary team (MDT) was formed to address the complex needs of the patient requiring HTPN, encompassing professionals from diverse fields such as nutrition, pharmacy, nursing, social work, home-based care, and palliative care. This collaborative approach ensured comprehensive and coordinated care, optimizing patient outcomes and overall well-being.

The first step in the process was the formation of a checklist to ensure the implementation of the program and the safe transition of that patient from hospital to home. We called it the home TPN checklist (Table 1).

**Table 1:** Home total parenteral checklist MDT, multidisciplinary team; TPN, total parenteral nutrition; N/A, not available; CHF, congestive heart failure; IV, intravenous; HPN, home parenteral nutrition

Topic	Checklist	Yes	No	N/A
Indication and appropriateness to start home TPN	The patient cannot meet the nutritional requirement by oral or enteral intake and has an approved indication for initiating TPN			
	The patient is able to receive TPN outside an acute care setting			
	There is clear documentation by the physician for the need of TPN			
	The patient is clinically stable before discharge			
	No significant renal failure, hepatic failure, and CHF			
	Diabetes under good control			
	The presence of active venous access			
	Intact volume status			
	Not at risk of refeeding syndrome			
	Ensure that the patient has appropriate home environment prior to discharge that is in compliance with medication storage policies and procedure			
	Availability of water, fridge, and telephone; safety issues; and any traveling plans			
Parenteral access	The patient has central IV catheter for TPN administration			
	The patient has infusion pump for administering TPN upon discharge			
Education	The patient has a competent nurse for administering TPN			
	Ensure proper training on pump use and care, catheter care, and recognizing common problems			
	The patient and family members are aware about their involvement in daily care			
Nutritional requirements	Appropriate caloric intake has been planned and agreed on with the physician and dietician			
Other IV medications	Other IV medications (if available) have no potential interaction with TPN			
Follow-up plan (monitoring of PN efficiency and nutritional status)	The MDT shall create a patient treatment plan prior to the patient's discharge from the hospital.			
	Appointment date/schedule			
	It should be appropriate with the TPN renewal date			
	Parenteral nutrition (PN) regimen			
	Daily nutrition log			
	Handling emergency situation and who to contact			
	The patient's needs including equipment and requisite medical supplies/consumables, visit protocol, duration, treatments, and safety and assessment timeframe have been met			
	The assigned physician is responsible for renewing the TPN order based on the follow-up appointments			
Insurance coverage	The patient is eligible for insurance coverage			
	Billing and reimbursement of the home healthcare shall be in accordance with standard provider contract and discussed with the pharmacy coder			
	Monthly billing is available to be submitted for insurance approval			
Nutrition support team	The MDT is available to facilitate patient transition of care			
Physician-dietician-pharmacist-nurse	Ensure that interdisciplinary teams engage in the assessment and provision of HPN			
	The individual care plan is made for the patient			
	Contact details are available or the post-discharge healthcare service/homecare			
	Contact details for the inpatient pharmacy had been provided to the patient/caregiver for any urgent inquiry about TPN			
	Ensure a proper TPN administration schedule that works for the patient's status			
	The time of TPN pickup is agreed upon between the pharmacy and the caregiver			
	The original prescription form after being signed by the doctor is retained in the patient's original file			
	A copy of the latest TPN prescription is issued to the patient upon dispensing			
	A copy of the patient TPN prescription is filed in the inpatient pharmacy			

Implementing the checklist began with staff training, which involved educating physicians, dieticians, pharmacists, and nurses on their respective roles in the patient's care. They were taught about the criteria for patient eligibility, proper handling of equipment, and managing potential complications. Next, patient and caregiver education took place, with an emphasis on understanding their responsibilities in daily care and familiarizing them with emergency contacts.

Once the patient was discharged, the multidisciplinary team (MDT) provided ongoing monitoring and support. Regular meetings were held to discuss the patient's status, adjust the HTPN order as needed, and ensure adherence to the treatment plan. Throughout the process, the MDT remained available for the patient and caregivers, addressing any concerns or inquiries that arose. This comprehensive approach ensured a seamless transition to home TPN and facilitated the best possible care for the patient.

## Discussion

The checklist for starting a palliative care patient on home total parenteral nutrition (TPN) covers various aspects, including patient eligibility, necessary equipment and support, and follow-up plans. To be eligible, the patient must have a clear need for TPN, be clinically stable, have an appropriate home environment, and have insurance coverage. Necessary equipment and support include a central intravenous catheter (peripherally placed central catheter), an infusion pump, and a competent nurse. The patient and family members should be involved in daily care and be informed of emergency contacts. A multidisciplinary team (MDT) must create a treatment plan that outlines appointments, nutrition regimens, and support resources. Regular meetings should be held to monitor the patient's progress, and all relevant contact information must be provided to the patient upon discharge. Documentation, such as a letter from the primary physician, a nutrition log sheet, and follow-up appointment schedules, should also be given to the patient upon discharge.

Total parenteral nutrition (TPN) is a life-sustaining treatment for patients who cannot maintain adequate nutrition through the gastrointestinal tract. It is a complex therapy that requires careful management and coordination of care. In the context of palliative care, TPN can be administered at home, providing patients with the comfort and familiarity of their own environment. However, initiating home TPN in palliative care patients involves several considerations, including patient eligibility, necessary equipment and support, and follow-up plans.

Patient eligibility is a crucial factor in determining the success of home TPN. According to a study by O'Flynn et al., patients receiving inpatient parenteral nutrition (PN) had a variety of indications, including short bowel syndrome, intestinal obstruction, and severe malabsorption [7]. These conditions often necessitate TPN as patients are unable to maintain adequate nutrition through the gastrointestinal tract. In the context of palliative care, patients must also be clinically stable and have an appropriate home environment to support the administration of TPN. Furthermore, the importance of careful patient selection, particularly in patients with advanced or relapsed cancer, is more challenging [8]. The high morbidity rates and low overall survival associated with these patients underscore the need for a supportive home environment and comprehensive care.

The necessary equipment and support for home TPN include a central intravenous catheter, an infusion pump, and a competent nurse. The costs associated with delivering home TPN interventions can be significant [9]. Our study underscores the importance of considering the financial implications of home TPN, including the costs of necessary equipment and support, and insurance coverage being an important factor in facilitating home TPN.

A multidisciplinary team (MDT) is essential in creating a comprehensive treatment plan for patients on home TPN. Carli et al. emphasize the importance of a multimodal program involving exercise, nutritional, and psychological interventions for patients undergoing colorectal cancer resection [10]. This approach could be adapted for palliative care patients starting on home TPN, emphasizing the need for a multidisciplinary team to create a comprehensive treatment plan. Regular meetings should be held to monitor the patient's progress, and all relevant contact information must be provided to the patient upon discharge.

## Conclusions

In conclusion, this study successfully implements an innovative, multidisciplinary checklist for managing elderly palliative care patients on home total parenteral nutrition (HTPN) in Al Ain, Abu Dhabi. The collaborative approach adopted by the multidisciplinary team, coupled with comprehensive staff training, patient and caregiver education, and ongoing monitoring and support, facilitated the seamless integration of HTPN into the patient's care plan. The positive outcomes observed in this case underscore the potential of such tailored interventions to bridge the existing gap in HTPN implementation within the region, thus improving the quality of life and overall well-being of elderly patients requiring specialized nutrition support.

Future research should focus on expanding the application of this multidisciplinary checklist approach to a larger cohort of patients, with the aim of elucidating its broader implications for healthcare practice and policy. Moreover, evaluating the cost-effectiveness and impact on healthcare resources of such interventions would provide valuable insights for decision-makers and stakeholders. By fostering a culture of innovation and collaboration in managing elderly patients requiring HTPN, we can strive to deliver the highest standards of care and contribute to the ongoing advancement of palliative care in Al Ain, Abu Dhabi, and beyond.
